# Draft Genome Sequence of *Enterobacter cloacae* Strain S611

**DOI:** 10.1128/genomeA.00710-14

**Published:** 2014-12-11

**Authors:** Dongping Wang, Cliff S. Han, Armand E. K. Dichosa, Cheryl D. Gleasner, Shannon L. Johnson, Hajnalka E. Daligault, Karen W. Davenport, Po-E Li, Elizabeth A. Pierson, Leland S. Pierson

**Affiliations:** a Bioscience Division, Los Alamos National Laboratory, Los Alamos, New Mexico, USA; b Department of Plant Pathology and Microbiology, Texas A&M University, College Station, Texas, USA; c Department of Horticultural Sciences, Texas A&M University, College Station, Texas, USA

## Abstract

We report draft genomes of *Enterobacter cloacae* strain S611, an endophytic bacterium isolated from surface-sterilized germinating wheat seeds. We present the assembly and annotation of its genome, which may provide insights into the metabolic pathways involved in adaptation.

## GENOME ANNOUNCEMENT

*Enterobacter cloacae* comprise a heterogeneous species that include many agriculturally beneficial strains. Strains of *E. cloacae* are rapid colonizers of the plant spermosphere and are often used to control fungal pathogens of crops and vegetables (1). Currently, no seed-borne *E. cloacae* genome has been sequenced.

We announce the draft genome of *E. cloacae* strain S611, isolated from surface-sterilized germinating wheat seeds. The isolated strain was able to rapidly proliferate in the wheat spermosphere. Comparative analysis of this genome with other related *E. cloacae* genomes will provide more insight into specific traits related to host adaptation.

Genome sequencing was performed using an Illumina MiSeq platform at the Bioscience Division, Los Alamos National Laboratory (2). Briefly, a modified TruSeq DNA Sample Prep version 2 and MiSeq sequencing protocol were used during the sequencing process (Illumina, San Diego, CA). Genomic DNA from strain S611 was isolated using the DNeasy miniprep kit (Qiagen, Hilden, Germany). The genomic library was constructed using 1 µg of genomic DNA fragmented using a Covaris E210 instrument. Fragments of 400 to 500 bp were extracted from agarose gels and used to construct a library. The library was amplified for 10 PCR cycles and quantified using the Qubit dsDNA HS assay, the Bioanalyzer High Sensitivity chip, and qPCR.

Filtered genomic sequences were assembled *de novo* using SPAdes (3), wherein 54 contigs with an average genome coverage of 87 (76–500) were obtained. The assembled data were annotated using an Ergatis workflow manager (4). Strain S611 has a genome of 4,437,247 bp, which is similar to other reported *E. cloacae* genomes and contains 3,996 putative protein-coding and 82 RNA-coding genes. Sequence analysis of two sigma factors, *rpoB* and *rpoD*, revealed the highest similarity (100% and 99% for *rpoB* and *rpoD*, respectively) with those from *Enterobacter* sp. strain SP1, an endophytic nitrogen-fixing bacterium isolated from sugarcane in China (5). Interestingly, genes encoding a putative type III secretion apparatus (accession nos. ESS59613 and ESS59860) are more similar to the cellulose-degrading bacterium *Pantoea* sp. SL1_M5 (6). The strain S611 genome harbors large numbers of genes encoding sugar and amino acid transporters, indicating its dependence on host-plant-derived nutrients. The genome also contains genes involved in flagella, type IV pili, and exopolysaccharide production, which may contribute to its long-term interactions with plants.

### Nucleotide sequence accession number.

The *E. cloacae* strain S611 whole-genome shotgun project was deposited at DDBJ/EMBL/GenBank under the accession number AXOM00000000.

## References

[1] Nelson EB 2004 Microbial dynamics and interactions in the spermosphere. Annu. Rev. Phytopathol. 42:271–309. 10.1146/annurev.phyto.42.121603.131041. 15283668

[2] Wang D Han CS Dichosa AEK Gleasner CD Johnson SL Daligault HE Davenport KW Li P-E Pierson EA Pierson LS III 2013 Draft genome sequence of *Pseudomonas putida* strain S610, a seed-borne bacterium of wheat. Genome Announc. 1(6):e01048-13. 10.1128/genomeA.01048-13. 24371199 PMC3873609

[3] Bankevich A Nurk S Antipov D Gurevich AA Dvorkin M Kulikov AS Lesin VM Nikolenko SI Pham S Prjibelski AD Pyshkin AV Sirotkin AV Vyahhi N Tesler G Alekseyev MA Pevzner PA 2012 SPAdes: a new genome assembly algorithm and its applications to single-cell sequencing. J. Comput. Biol. 19:455–477. 10.1089/cmb.2012.0021. 22506599 PMC3342519

[4] Orvis J Crabtree J Galens K Gussman A Inman JM Lee E Nampally S Riley D Sundaram JP Felix V Whitty B Mahurkar A Wortman J White O Angiuoli SV 2010 Ergatis: a web interface and scalable software system for bioinformatics workflows. Bioinformatics 26:1488–1492. 10.1093/bioinformatics/btq167. 20413634 PMC2881353

[5] Zhu B Chen M Lin L Yang L Li Y An Q 2012 Genome sequence of *Enterobacter* sp. strain SP1, an endophytic nitrogen-fixing bacterium isolated from sugarcane. J. Bacteriol. 194:6963–6964. 10.1128/JB.01933-12. 23209221 PMC3510633

[6] Adams AS Jordan MS Adams SM Suen G Goodwin LA Davenport KW Currie CR Raffa KF 2011 Cellulose-degrading bacteria associated with the invasive woodwasp *Sirex noctilio*. ISME J 5:1323–1331. 10.1038/ismej.2011.14. 21368904 PMC3146269

